# What are cultural attractors?

**DOI:** 10.1007/s10539-017-9570-6

**Published:** 2017-03-17

**Authors:** Andrew Buskell

**Affiliations:** Department of History and Philosophy of Science, University of Cambridge, Free School Lane, Cambridge, CB2 3RH UK

**Keywords:** Cultural evolution, Cultural epidemiology, Cultural attractor theory

## Abstract

Concepts from cultural attractor theory are now used in domains far from their original home in anthropology and cultural evolution. Yet these concepts have not been consistently characterised. I here distinguish four ways in which the cultural attractor concept has been used and identify three kinds of factors of attraction typically appealed to. Clarifying these explanatory concepts identifies problems and ambiguities in the work of cultural epidemiologists and commentators alike.

## Introduction

Cultural attractor theory (hereafter; CAT) is one of several approaches within cultural evolutionary theory with the aim of explaining how ideational variants change in their distribution and form over time (more on this below). Within cultural evolutionary theory, CAT is distinguished by its *transformative approach*, holding that the content of variants are transformed during the acquisition process (Sperber 1985, 1996).

The central explanatory concept of CAT is that of *cultural attractors*. Cultural attractors are theoretical posits that capture the way in which certain ideational variants are more likely to be the outcome of transformations than others. Insofar as these transformations are systematic across members of a population, CAT can be used to explain changes in the distribution and form of ideational variants in a population over time. It is in this way that CAT has been used in anthropology to explain the ubiquity and diversity in cross-cultural beliefs in the supernatural (Boyer 2001), in psychology to explain why some sentences and stories are better remembered than others (Heath et al. 2001; Norenzayan et al. 2006; Mesoudi and Whiten 2008), in linguistics to explain the evolution of grammatical structures (Scott-Philips 2014), and in cultural evolution to provide distribution explanations (Claidière and Sperber 2007).

The aim of this paper is not to evaluate the empirical merits of CAT. Instead, I here aim at describing and clarifying the concepts it employs. Of course the empirical merits of a theory go hand-in-hand with its conceptual tools. Yet in elucidating and clarifying the nature of these tools, I here take much of what CAT researchers (hereafter; ‘cultural epidemiologists’)^1^ have to say about the empirical payoffs of their approach at face value.

The paper is organised as follows. In the next section, I introduce a distinction between transformative and preservative approaches to studying cultural evolution. What is important about this distinction is not, or not just, a difference between the fidelity of the underlying processes themselves—but the way in which change in the underlying content of vehicles is brought about. While transformative approaches take it that change involves the (re-)constructing activities of systems, preservative approaches take it that change involves error or noise in a copying process. On the back of this distinction, I show why it is wrong to identify CAT’s transformative approach with concepts drawn from preservative approaches.

However, much of the action takes place in the middle sections where I analyse cultural attractors and factors of attraction. As I show, there are four distinct conceptualisations of the cultural attractor concept, with such cultural attractors (however conceptualised) being the result of a number of distinct kinds of underlying causes—or *factors of attraction*. I argue that by isolating these distinct notions of cultural attractors and highlighting the various kinds of factors of attraction, we are afforded a greater conceptual grip on the central commitments of CAT.

## Preservative and transformative approaches

The study of cultural evolution has multiple aims. One important aim is to describe and explain the means by which ideational variants (things like norms, beliefs, skills, and the like) and their behavioural expressions (artefacts, institutions, social arrangements and the like) are acquired and distributed through populations. The use of the term ‘ideational’ is meant to focus attention on the mental or cognitive—and as I use the term, ‘ideational variants’ pick out mental representations whose contents are expressed in behaviour (Sperber 1996; Mesoudi 2011). In a rough and ready way, one can understand the contents of these representations as ‘recipes for action’.

Important for later discussion is a notion of similarity between ideational variants. When I speak about the similarity between variants I have in mind something like the following: when individuals have similar representational contents (similar ideational variants) this similarity leads to the expression of similar behaviours. Of course, the notion of ‘similarity’ is fraught, and filling in precisely what is meant by ‘similar content’ or ‘similar behaviour’ and how the two are to be mapped on to one another will be complex manner (Charbonneau 2015), but for current purposes this intuitive characterisation will suffice.

As I have said, one aim of the study of cultural evolution is to provide distribution explanations. These explanations account for how and why some ideational variants [v_1_, v_2_, …, v_n_] at some distribution [d_1_, d_2_, …, d_n_] engender, at some latter time-step, the distribution [d’_1_, d’_2_, …, d’_n_]. So given the following set of ideational variants [*a*, *b*, *c*] with frequency distributions of [20, 50, and 30%] at time *t*_1_, a distribution explanation would account for how and why this distribution has changed at time *t*_2_ to [10, 30, 60%].

The complexities and contingencies involved in human culture frustrate a one-size-fits-all approach to generating cultural evolutionary distribution explanations. As a result, cultural evolutionary researchers have adopted a strategy of piecemeal modelling, employing multiple idealised and abstracted models. Such models typically examine the effect of a single causal factor on distributions of ideational variants. The has produced a literature rich in models that examine the impact of specific psychological, sociocultural, and demographic factors on distributions of ideational variants over time (e.g. Cavalli-Sforza and Feldman 1981; Boyd and Richerson 1985; Henrich and Boyd 2002). To take one example, Henrich (2001) has argued that learning influenced by a conformist bias—here, a bias towards acquiring an ideational variant at a disproportionately higher frequency than the prevalence of that behaviour in the population—generates a recognisable ‘s-shaped’ sigmoidal diffusion curve under a range of parameterisations.^2^ The overall aim of this modelling strategy is to produce a set of such models that can be combined (albeit in complex ways) to generate distribution explanations for complex, real-world scenarios (Boyd and Richerson 1985).

One can identify two approaches to modelling distribution dynamics in the cultural evolutionary literature. The first takes the process of acquiring ideational variants to be *preservative*, where individuals acquire variants with fidelity from some pre-existing template or model (Sterelny and Griffiths 1999; Sterelny 2006). The second approach takes the process of acquisition to be a *transformative*, where individuals reconstruct what is acquired during the acquisition process (Sperber 1996).^3^

The ‘modern synthesis’ in evolutionary biology has tended to view biological inheritance as, at root, preservative (Pigliucci and Müller 2010). According to standard characterisations, the acquisition of variants is explained by pointing to a template-copying procedure, one that creates copies of parental DNA that are subsequently passed on to offspring. Nonetheless, the explanatory emphasis on preservation is compatible with integral sources of change and disruption. Mutation is just such an integral process that introduces new variation into a population by changing the content of variants (phenotypes) in virtue of modifications to the underlying vehicles of such variants (genome sequences). Yet while mutation is integral to evolution, the modern synthesis has not taken it to represent an interesting phenomenon. Instead, they have foregrounded the explanatory relevance of preservative processes and selection, while concomitantly reducing the role of mutation to a background rate of change, or error (cf. McShea and Brandon 2010).

As I see it, the crucial difference between transformative and preservative approaches turns on how each conceives of transmission and change. The preservative approach holds that changes in content are introduced when error or noise in a copying process introduces new variation—whether for better or for worse. The transformative approach, by contrast, holds that changes in content occurs as individuals (re-)construct variants given the operation of some (re-)constructing system. In virtue of the variable nature of such a system, the same signal or stimuli may lead to radically different reconstructions.^4^ In biology this kind of transformative approach is championed by proponents of dynamical systems theory who argue that ontogeny (which here includes a variety of reliable environmental features) forms a background system that flexibly interprets signals and stimuli in the production of phenotypes (Griffiths and Gray 1994; Oyama 2000).

This distinction between preservative and transformative approaches offers an illuminating perspective on the way in which cultural evolution has been conceived and modelled by different researchers. CAT emphasises the explanatory role of the transformative approach, where reconstructive processes during learning lead to variation in the content of ideational variants (Sperber 1996). By contrast, other approaches assume a preservative approach, where ideational variants are acquired with fidelity (e.g. Boyd and Richerson 1985; Henrich 2001, 2016). However, the consensus today seems to be that both kind of approaches are necessary to a full understanding of cultural evolution (e.g. Henrich and Boyd 2002; Henrich 2004; Sperber and Claidière 2008; Morin 2015), and that theoretical differences between the approaches may result from the spatiotemporal grain at which their respective investigations tend to take place (Acerbi and Mesoudi 2015). Indeed, I think that most cultural evolutionary researchers would agree with the statement of Sperber and Claidière (2008) when they write that the acquisition of variants “always involves a combination of preservative and constructive [that is, transformative] processes.” (p. 287. Cf. Sterelny 2001; Henrich et al. 2008).^5^

Nonetheless, despite this consensus position, it is incorrect to collapse these two approaches. On this point, distinguishing between transformative and preservative approaches can help to explain the resistance that cultural epidemiologists have had to the identification of their approach with what Peter Richerson, Robert Boyd and Joseph Henrich call ‘direct biases’ (i.e. Boyd and Richerson 1985) or ‘content-based’ biases (Richerson and Boyd 2005; Henrich et al. 2008). Such content-based or direct biases are those that make individuals “more likely to learn or remember some cultural variants based on their content. Content-based bias can result from calculation of costs and benefits associated with alternative variants, or because the structure of cognition makes some variants easier to learn or remember.” (Richerson and Boyd 2005, p. 69) But this already assumes an underlying preservative approach, with content-based biases acting as a selective process either during memory consolidation or by means of direct comparison (Boyd and Richerson 1985). Such selective processes determine whether and to what extent one ideational variant, acquired with fidelity, is retained over time. But as Sperber and Claidière (2008) note, these biases do not involve the transformation of the “contents of cultural variants [instead] they affect only their frequency […] in the end some variants are more often retained than others.” (p. 287).

In a similar manner, it is also wrong to identify the transformative approach with guided variation (e.g. Mesoudi et al. 2013; Acerbi and Mesoudi 2015). According to Boyd and Richerson’s original formulation, guided variation occurs when individuals learn from a model, and then “modify their initial phenotype according to [a] learning rule.” (p. 95. Cf. Boyd and Richerson 1984; p. 430; Henrich 2001). For Mesoudi, this modification is whatever “[c]auses cultural evolution to shift toward inferential prior knowledge.” (Mesoudi et al. 2013, p. 197)^6^ This does sound very similar to a transformative approach, and Mesoudi assumes that “[g]uided variation resembles what cognitive anthropologists have called “cultural attraction”” (Mesoudi et al. 2013, p. 197).

However, this too should be resisted by cultural epidemiologists. This is for the simple reason that guided variation assumes that initial learning can be characterised in a preservative manner (the initial learning of a ‘phenotype’ from some cultural model) that is only subsequently transformed. Furthermore, guided variation is construed in such a manner that this subsequent transformation is something that can take place over extended periods of time, as individuals tinker, experiment, and change their acquired variants (Boyd and Richerson 1985). But cultural epidemiologists have been forceful in arguing that transformational processes are at work *during* the acquisition process (Sperber 1996, 2000). So the transformative approach should not be identified with content-biases, nor guided variation.

As a final comment, it is important to note that both transformative and preservative approaches can conceptualise and characterise processes that lead to systematic and directional changes in the distribution of variants in a population (Boyd and Richerson 1985; Henrich and Boyd 2002; Claidière and Sperber 2007). As in biological evolution, in cultural evolution high-fidelity processes can lead to the differential growth of one type of variant over another. Yet the transformative approach too can explain directional population-level effects. It can do so under the assumption that individuals in a population transform the content of variants in similar ways. Yet, while these two kinds of explanations may account for similar kinds of directional effects, they are likely to appeal to very different kinds of underlying causal processes and grains of analysis.

## Cultural attractors and factors of attraction

As I have argued, cultural epidemiologists adopt a transformative approach to studying culture (e.g. Sperber 1985, 1996). Practically, this means that CAT models assume distributions of ideational variants are “largely determined by the transformation of culture.” (Claidière and André 2011 p. 13) Central to this transformative approach is the concept of cultural attractors. Cultural attractors are part of an explanatory endeavour “[aimed] at explaining the relative prevalence and stability of cultural contents.” (Claidière and Sperber 2007, p. 91) In other words, cultural attractors are involved in providing distribution explanations. But what are cultural attractors? As Dan Sperber writes:

[An] attractor, as I have characterized it, is an abstract, statistical construct, like a mutation rate or a transformation probability. To say that there is an attractor is just to say that, in a given space of possibilities, *transformation probabilities form a certain pattern*: they tend to be biased so as to favour *transformations in the direction of some specific point*, and therefore *cluster at and around that point*. An attractor is not a material thing; it does not physically ‘attract’ anything. To say that there is an attractor is not to give a causal explanation; it is to put in a certain light what is to be causally explained: namely, a distribution of items and its evolution, and to suggest the kind of causal explanation to be sought: namely, the identification of genuine causal factors that bias micro-transformations.” (Sperber 1996, pp. 111–112, italics added)

It is crucial to note that there are a number of unresolved ambiguities in this characterisation. Should attractors be understood as descriptions of individual tendencies to transform variants in patterned ways? Or by contrast, should cultural attractors be understood as ‘specific points’ in some abstract ‘variant-space’ around which variants tend to cluster? And what are these factors of attraction—the underlying casual factors that generate transformation probabilities?

In what follows, I discuss the various, and sometimes contradictory, ways in which cultural attractors and factors of attraction are employed in CAT. I first identify four distinct ways in which the cultural attractor concept has been characterised, and suggest that the tendencies they purport to capture need not lead to concordant distributions. Second, I examine factors of attraction, identifying three distinct kinds of factors. Here I suggest that some of these factors of attraction may require further philosophical and theoretical attention.

## Four characterisations of the cultural attractor concept

As Sperber argues in the quote above, cultural attractors are a statistical construct, like a mutation rate or transformation probability. These he thinks, generate a particular pattern. In turn, this pattern tends “to be biased so as to favour transformations in the direction of some specific point, and therefore cluster at and around that point.” (Sperber 1996, p. 112) But as I noted above, this characterisation is ambiguous. Are cultural attractors a particular pattern—an aggregate of transformation probabilities—or are attractors wherever variants happen to cluster?

It is important to note that these two conceptualisations need not coincide. Patterns of transformation probabilities can be unevenly distributed across a variant landscape, and such landscapes can have ‘high’ and ‘jagged’ peaks. Furthermore, transformations can be ‘jumpy’: reconstruction can be such that one adopts a radically different variant from one’s model. Indeed, one can imagine any number of possible transformation patterns that are unlikely to generate clustering around a particular variant. Sperber’s suggestions to the contrary, there is no straightforward conceptual link between patterns of transformation and clustering of variants.

But these are not the only conceptualisations of cultural attractors. A distinct characterisation is given in a recent paper by Claidière et al. (2014). Here, “processes […] may *tend to transform different inputs in similar ways* (rather than randomly), and in doing so cause the outputs to *tendentially converge upon particular types*, called attractors.” (p. 4, italics added) Here too there is an ambiguity between understanding attractors as a tendency of individuals to transform inputs in patterned ways, and as a tendency for variants to converge on a particular type. Nonetheless, Claidière et al. (2014) define attractors as “any type whose relative frequency tends to increase over time.” (p. 5)

Finally, consider an intriguing cultural feature central to CAT. Cultural epidemiologists often talk about ‘macro-level stability’, the idea that “cultural information is relatively stable within whole populations and often across generations.” (Claidière and Sperber 2007, p. 91. Cf. Sperber 1996, 2000). Indeed, Claidière and Sperber (2007) assert that attractors only change over “historical time, that is, slowly enough to uphold the relative stability of culture.” (p. 92)^7^ This is allied with a position where “cultural contents owe much of their stability to the directionality of constructive psychological processes.” (Sperber and Claidière 2008, p. 289). For CAT, the stability of culture is a key explanandum.

An intuitive way of capturing this stability is to wed it to the notion of *equilibria states*. Cultural attractors might be identified with the distribution at which various ideational variants come to settle. This seems to be how cultural attractors are in fact identified by Claidière et al. (2014). Claidière et al. are concerned with articulating a sparse modelling strategy they term *evolutionary causal matrices*. Without getting too deep into the complexities of this modelling strategy, we can characterise these matrices as simple recipes for determining how different types of ideational variants change in absolute number (and, by extension, relative frequency) over time. In the examples considered by Claidière et al., these matrices settle into a stable frequency distributions—more specifically, stable global equilibria.

It should be noted that Claidière et al. (2014) never explicitly identify cultural attractors with equilibria. However, it is clear from their arguments that it is only when the relative frequency of variants enters into a stable equilibrium that one can adjudicate whether or not there are attractors. Arguments from Acerbi and Mesoudi (2015) suggest that this is how they too take Claidière et al. (2014). Acerbi and Mesoudi argue that what they call the ‘extended’ interpretation of attraction holds that ““attraction” is synonymous with any directional change in cultural evolution” (Acerbi and Mesoudi 2015, p. 488). Insofar as *any* directional change (that is, change in the frequency of ideational variants) will end up stabilising at global equilibria, this extended interpretation holds that such equilibria points are cultural attractors (Acerbi, pers. comm.). Thus, irrespective of whether or not Claidière et al. (2014) believe that cultural attractors can be identified equilibria points, their remarks and modelling approach suggest as much. I take it, then, that it is thus worthwhile to identify and analyse this position, if only for subsequent use as a foil for cultural epidemiologists to clarify their position.

So ‘cultural attractor’ is a statistical concept, picking out some tendency of a population, which is in turn the result of a number of underlying factors (more on this below). Here I have argued that cultural epidemiologists have missed ambiguities in their characterisations of this tendency. Indeed, I suggest that we can distinguish four distinct conceptualisations, or notions, of the cultural attractor concept from the analyses above. These four notions are tendencies: (1) for individuals to transform variants in patterned ways; (2) for variants to cluster around a specific point; (3) for a variant (or variants) to increase in relative frequency, and; (4) for variants to settle at some equilibrium point. From here on, I will refer to these simple as TRANSFORMATION PATTERN, CLUSTER POINT, INCREASING FREQUENCY, and EQUILIBRIUM POINT respectively.^8^, ^9^

These different notions of the cultural attractor concept can be clearly distinguished. Useful in this endeavour is Claidière and Sperber’s (2007) model of smoking behaviour. This is a simple model that examines the frequency of various behaviours in a population; here, how many cigarettes one smokes per day. In Claidière and Sperber’s models, there are thirty-one possible smoking behaviours: smoking zero cigarettes per day up to smoking thirty cigarettes per day (the authors assume that individuals only smoke whole cigarettes). In this model each time-step involves a new cohort of smokers replacing the old, but only after individuals in this new cohort pick a model from the older generation to learn from. This separates out the ‘sampling’ of models from the ‘learning’ from such models, and allows Claidière and Sperber to evaluate the impact of both *sampling biases* (biases that impact who individuals learn from, what the authors call *cultural selection*) and the *force of attraction* (the impact of transformative processes during learning) on variant distributions.

**Fig. 1:**
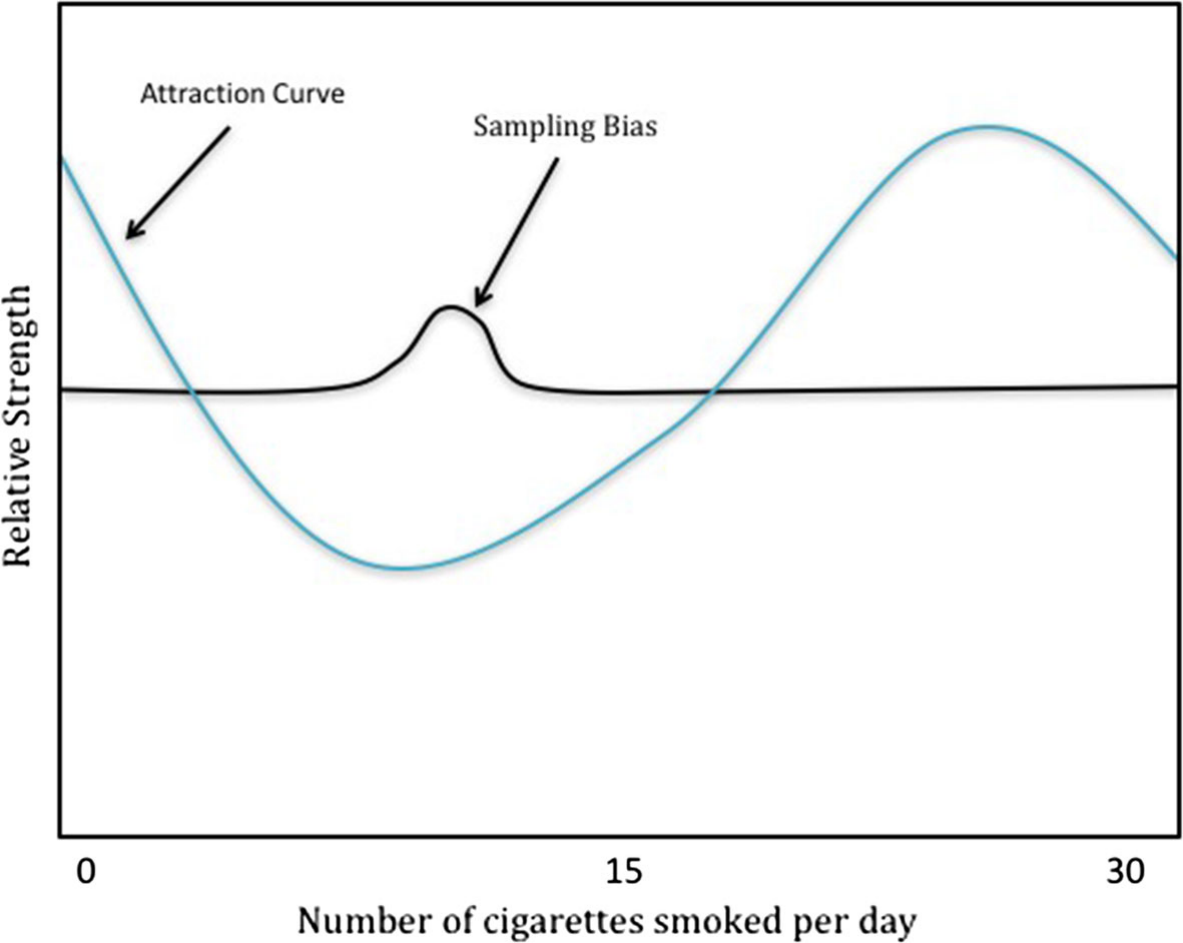
The relative strength of the force of attraction and sampling biases (adapted from Claidière and Sperber 2007)

In all of Claidière and Sperber’s models, sampling biases cause individuals to preferentially learn from individuals who smoke ten cigarettes a day. Following the choice of a cultural model, individuals learn how many cigarettes to smoke. This learning is transformative, with individuals reconstructing variants in a way influenced by the force of attraction. This influence is represented by a mathematical function, what the authors call the *attraction curve*, one whose strength peaks at five and twenty-five cigarettes per day (Fig. 1). For Claidière and Sperber, higher strength at distinct points of the attraction curve signals a greater ‘pull’ on individuals to transform variants toward the attractor peaks. Thus, to say that the strength of an attractor increases towards a peak means that the likelihood of adopting the behaviour of smoking five cigarettes or twenty-five cigarettes increases disproportionately as individuals learn from models close to these peak values.

It is straightforward to identify TRANSFORMATION PATTERN with the mathematical description of the force of attraction captured by the attraction curve. After all, this function describes the tendency of the population to reconstruct variants in certain patterned ways. It describes, in other words, the aggregation of all the transformation probabilities in a population, with variants that individuals are more likely to reconstruct having higher ‘strength’. This identification is supported by the scenario wherein the strength of sampling bias (the bias to disproportionately select certain individuals as models) is set to zero, and as a result, individuals randomly pick models to learn from. Under such parameters, the frequency distribution of smoking behaviour comes to settle on values reflecting the attraction curve, with the greatest frequency of behaviours clustered around the attraction peaks at five and twenty-five (Claidière and Sperber 2007, pp. 94–95). As Claidière and Sperber (2007) write: “If there was only attraction and no selection, we would expect after some time the distribution of smoking patterns to correspond to the attraction curve.” (p. 94) That is, the distribution would come to reflect the aggregation of transformation probabilities.

**Fig. 2:**
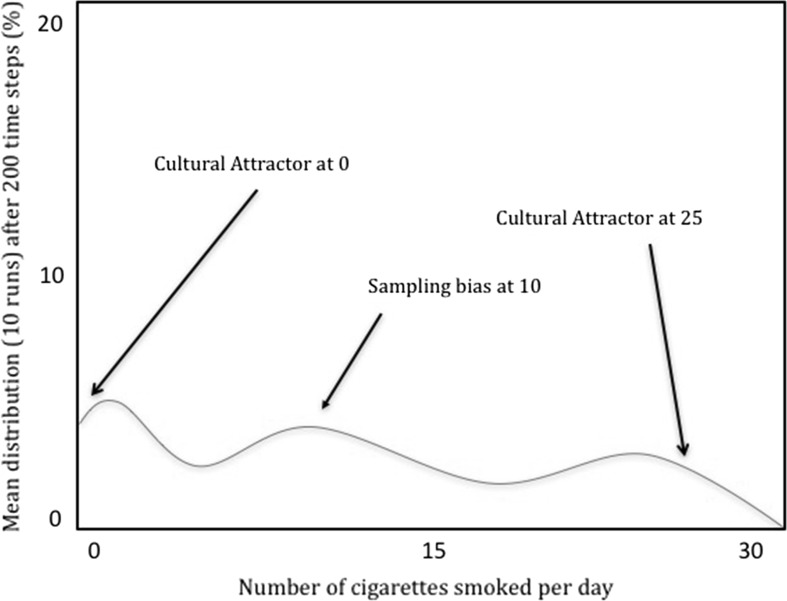
The mean distribution of variants after ten model runs of 200 generations with both sampling bias and the force of attraction. Note that variants do not cluster *at* (but do cluster *near*) the cultural attractor peaks, while they do cluster *at* the sampling bias peak (adapted from Claidière and Sperber 2007)

**Fig. 3:**
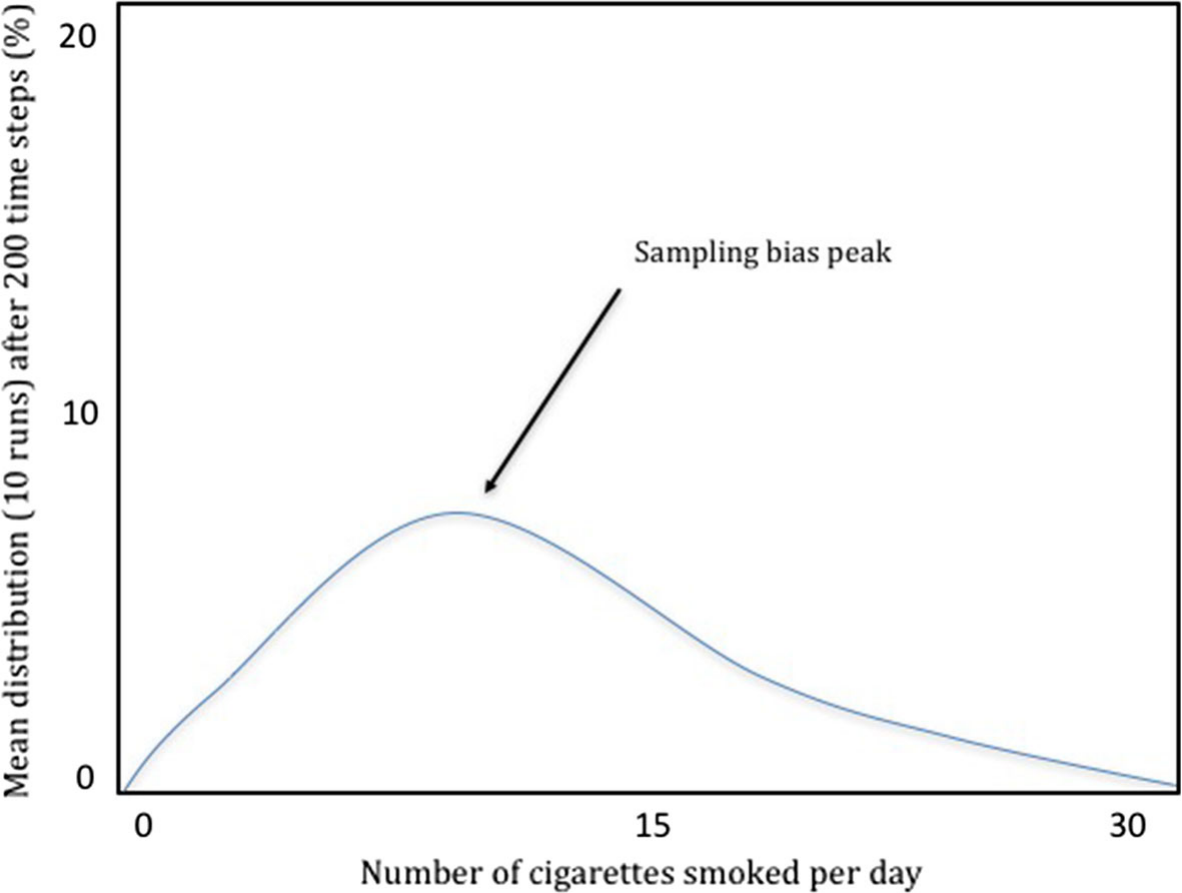
The mean distribution of smoking behaviour after ten model runs of 200 generations. Here smoking behaviour is clustered around the sampling bias peak of ten cigarettes smoked per day (adapted from Claidière and Sperber 2007)

Different parameterisations of Claidière and Sperber’s model, however, show how TRANSFORMATION PATTERN can pull apart from the other notions, notably, CLUSTER POINT and INCREASING FREQUENCY. To see why, consider what happens when sampling biases are reintroduced (Claidière and Sperber 2007, p. 108). When sampling biases are non-negligible, the distribution of variants is skewed towards ten, away from the attractor peaks of zero and twenty-five (Fig. 2). Indeed, by manipulating the strength of sampling biases, cultural attractors, and the initial distribution of variants, one can change which variants will have increased by the end of a model’s run—variants that might be far from the attractor peaks as described by the attraction curve. When the strength of sampling bias is the predominant factor, for instance, variants will come to cluster around ten cigarettes (Fig. 3). It seems clear, then, that neither CLUSTER POINT nor INCREASING FREQUENCY need coincide with TRANSFORMATION PATTERN.

So when Claidière and Sperber de-idealise their model, adding back in sampling biases, it demonstrates that at least three notions of the cultural attractor concept do not coincide. But what about EQUILIBRIUM POINT? How does it line up with the other notions?

Recall that EQUILIBRIUM POINT is associated with one of the central motivating issues for CAT, that transformations explain the *stability* of culture over time (Sperber 1996, 2000, 2001; Claidière and André 2011; Claidière et al. 2014; Morin 2015). I have suggested that at least one plausible way of cashing this out is in terms of stable equilibria into which variants settle. As such, EQUILIBRIUM POINT is not something that can be captured by a single run of a model. It is something that can only be identified by looking at a model under a range of different parameterisations and by performing analyses to determine where and under what parameters the variants of a model settle into a stable equilibrium state (e.g. Creanza and Feldman 2014).

With this in place we can now note that the same TRANSFORMATION PATTERN, that is, the same characterisation of how individuals transform variants, can generate a range of equilibria. As Claidière and Sperber (2007, pp. 107–109) show, by varying the relative strength of the force of attraction (the TRANSFORMATION PATTERN) to sampling bias, one can generate stable equilibria with very different distributions of variants.

The shifting of equilibria has implications for CLUSTER POINT and INCREASING FREQUENCY as well: while some parameterisations may lead to variants increasing in frequency or clustering when reaching the EQUILIBRIUM POINT, the same variants in other parameterisations may decrease in frequency or not involve clustering. EQUILIBRIUM POINT is not only distinct from TRANSFORMATION PATTERN but from CLUSTER POINT and INCREASING FREQUENCY as well.

The punchline of all these considerations is that the four statistical notions of the cultural attractor concept are each identified with distinct kinds of population-level tendencies. These tendencies may, in a variety of situations, generate distinct frequency distributions.

At this point, we might reasonably wonder: why is it that these varied notions of the cultural attractor concept have escaped the notice of cultural epidemiologists? To my mind there are two plausible answers. The first implicates idealisation: when forces other than attraction are negligible, the various notions of the attractor concept may coincide. That is, variants can settle into a stable distribution (EQUILIBRIUM POINT), where the variants that increase in relative frequency (INCREASING FREQUENCY) cluster together (CLUSTER POINT) in virtue of the shared, aggregate transformation probabilities of individuals (TRANSFORMATION PATTERN). In such an idealised circumstance, all four underlying tendencies may be concordant: they all lead to the same population-level outcome. But when de-idealised—for instance, when sampling biases are reintroduced and parameters are varied (as Claidière and Sperber do above)—these four tendencies may generate discordant frequency distributions. The issue here is that cultural epidemiologists may have predominantly theorised about cultural attractors in idealised circumstances, and this has hindered their ability to see the ambiguity in the concept being used.

Related to this is a second possibility, most forcefully expressed by Alex Mesoudi (2011). CAT lacks well-developed sets of formal models that operationalise the cultural attractor concept. The notable exceptions—Claidière and Sperber (2007) and Claidière et al. (2014)—are not put forward by the cultural epidemiologists as foundational models, but instead as instrumental tools to show that the ‘pull’ of cultural attractors (the ‘force of attraction’) need not straightforwardly combine with sampling biases (Claidière and Sperber), or that an abstract characterisation of attractors might generate similar dynamics to those seen in formal population genetics (Claidière et al.). So while it might sound as though CAT is grounded in formal concepts derived from dynamic systems theory, in fact, terms like ‘attraction’, ‘attractor’, and the like, are qualitative terms. Indeed, the arguments and methods of CAT remain predominantly qualitative. For Mesoudi, at least, the lack of work grounding these concepts in formal models may explain why cultural epidemiologists have had problems noticing ambiguity.

Mesoudi’s point has bite, and I hope that the reflections here spur cultural epidemiologists to produce further models, and to focus greater attention on how the cultural attractor concept should be operationalised. Nonetheless, one can resist the position implicit in some of Mesoudi’s assertions; that the only good work in cultural evolution is quantitative. Though formal modelling can make explicit the otherwise implicit assumptions of theories, one should be cautious in seeing all qualitative work as vague and valueless. Despite his occasionally tough rhetoric, I suspect Mesoudi (2011) would agree—both qualitative and quantitative work is valuable, and progress is often made when the two enter into a vigorous dialectic.

## Factors of attraction

The cultural attractor concept is statistical, picking out populational tendencies. These tendencies in turn are the aggregate result of many lower-level processes. These lower-level processes are what cultural epidemiologists call *factors of attraction.* But what are these factors of attraction? Here too, I suggest, we find a great deal of variation. In what follows, I distinguish three different sets of causal factors that have been discussed in the literature: *reconstructive learning*, *motivational factors*, and *ecological factors*. These sets may be invoked separately or together as the underpinnings of a particular cultural attractor, but at least as I construe these sets they are non-overlapping: psychological processes implicated in reconstructive biases are not identical with those implicated by motivational factors or ecological factors.

Let me begin by articulating the factors of attraction most often discussed by cultural epidemiologists (Sperber 1985, 1996, 2000, 2001; Claidière and Sperber 2007; Claidière et al. 2014). According to Sperber (1996), an important set of causal factors involves the “convergence of your affective and cognitive processes with those of many people towards some psychologically attractive type of views in the vast range of possible views.” (Sperber 1996, p. 106) Such ‘convergence’ also implicates one’s background knowledge (Sperber 1996). As Claidière and Sperber (2007) write, when “an individual acquires a new cultural item (e.g. a skill, a belief, or a norm), she never just copies the variant or variants she observes; rather, drawing on the information transmitted and her own background knowledge, inferential abilities, and interests, she constructs a variant of her own.” (p. 91) Let us call this set of processes, those that involve doxastic, affective, axiological, and cognitive influences on the inferences made during learning, *reconstructive learning*.^10^

Factors salient to reconstructive learning will vary according to the relevant contrast classes. As Sperber (1996) writes, such learning is “rooted in part in universal human psychology and in part in the local cultural context.” (p. 108) If this is the case, then all human individuals will to some extent share transformative processes in virtue of their shared evolutionary history. These processes might include those typically invoked by cultural epidemiologists, like ‘folk knowledge’, ‘folk biology’, ‘folk physics’, and, perhaps, agency detection (Sperber 1996; Atran 1998; Boyer 2001; Sperber and Hirschfeld 2004, 2007). But these deep psychological structures are not always the most salient explanatory factors. Across different human populations, individuals will be influenced by local norms and mores, the beliefs that they acquire as part of being raised in a particular culture, as well as more idiosyncratic differences rooted in personality and individual experience. Different contrast classes will here demarcate the relevant kinds of biases on the inferential processes occurring during learning.

Reconstructive learning is what Acerbi and Mesoudi (2015) call the ‘narrow notion’ of cultural attractors, one where “cultural transmission is a mainly reconstructive process” (p. 483) and involves “transformative, non-selective processes in cultural transmission.” (p. 488). Indeed, reconstructive learning is identified by Mesoudi (2011; Mesoudi and Whiten 2008; Mesoudi et al. 2013) with the experimental methodology of ‘transmission chains’ (Hoppitt and Laland 2013). These are experiments where agents (or groups of agents) transmit information ‘down the chain’ from one individual to the next, not unlike the children’s game ‘telephone.’ This experimental paradigm can reveal transformational tendencies. For example, Kimmo Eriksson and Julie Coultas and the philosopher Shaun Nichols provide evidence that something like ‘core disgust’—where a strong negative affective reaction combining “(1) a sense of oral incorporation […]; (2) a sense of offensiveness; and (3) contamination potency” (Rozin et al. 2000, p. 640)—has a noticeable effect on the extent to which stories are modified and retained in memory (Eriksson and Coultas 2014) and the moral norms that are likely to be preserved in populations over time (Nichols 2002).

But while reconstructive learning and transmission-chain studies capture an important aspect of CAT, cultural epidemiologists often invoke other sets of causal factors besides reconstructive learning. These factors don’t fit neatly into Acerbi and Mesoudi’s ‘narrow notion’. Consider here Olivier Morin’s (2015) notion of *motivational attraction*. These are putative factors of attraction that “make us want to use or transmit” (p. 148) a particular variant. Morin’s chosen example involves coercion:

Legend has it that the Manchu haircut (a tight braid in the back behind a shaved head) had been adopted because it freed the field of vision of steppe raiders. Others say the braids could be used as a pillow to sleep on the rough. […] Either way, it was such a strong symbol for the Manchu Qing dynasty that it decided, upon its accession, to make it mandatory for all the emperor’s subjects, on pains [sic] of death.

Here it is more than reconstructive learning that explains the spread of the Manchu haircut: it is the threat of death! The ‘Qing edict’ here is implicated as a (particularly weighty) factor in individual risk/reward calculations, and the result of such calculations explains the widespread adoption of the Manchu haircut. Morin’s motivational attraction—or motivational factors of attraction—is thus identified with a range of (potentially) deliberative strategies for adopting and spreading certain variants.

Morin does not make a sharp conceptual distinction between reconstructive learning and motivational factors, but here is one possible means of doing so: motivational factors are implicated in biasing personal or sub-personal decisional processes as to *which* behaviour (from a behavioural repertoire) one should express in an appropriate situation. This characterisation makes clear that while motivational factors influence *which* behaviours one is motivated to produce, reconstructive learning influences *what* variant one will acquire during such learning, and *when* such variants are appropriate to produce.

Finally, cultural epidemiologists do not just speak about psychological factors. They also appeal to a wide variety of *ecological factors* of attraction (Sperber 1985, 1996, 2001; Claidière and Sperber 2007; Claidière et al. 2014; Acerbi and Mesoudi 2015). Just as there are natural barriers (oceans, mountain ranges) that in part determine the spread of diseases—so too, the thought goes, can ecological factors impact the spread of ideational variants. To take one example, if there is no clay in my local environment, I won’t be able to produce clay tablets for writing or throw pots (Sperber and Claidière 2008).

That the environment can play a role in frustrating or facilitating the spread of ideational variants is obvious. And if the term ‘factors of attraction’ picks out all the causal processes that may be relevant to the spread of ideational variants, then it will include such environmental and ecological causes. While this necessitates that CAT’s theoretical apparatus encompasses a wide range of phenomena—including extremely local and contingent events—I do not see this as much of an impediment. After all, models in evolutionary theory capture heterogeneous processes by grouping them into abstractly characterised processes such as drift or selection.

Nonetheless, drift and selection are taken to capture different kinds of heterogeneous processes and their effects on populations. So too might we think that ecological factors of attraction might be organised and taxonomised into distinct kinds of causal processes. There are scattered remarks throughout the CAT literature suggesting such distinct kinds. Sperber (1996, p. 113) identifies local culture, ancestral selection pressures, the material make-up of artefacts, and the ‘medium of transmission’ as distinct kinds of ecological factors of attraction. Morin’s (2015) suggests that *accessibility* to the public productions of culture is another. Finally, Heintz and Claidière (2015, p. 804) suggest that access to *resources* (rather than access to public expressions of ideational variants) and “multiple aspects of the natural environment” are involved in the production of culture.

Yet as these few remarks suggest, cultural epidemiologists have not focused their energies on taxonomising and characterising ecological factors of attraction. Indeed, much of the empirical and conceptual work in CAT is targeted at psychological factors of attraction. This is unfortunate, not least because these ecological factors of attraction seem to work at various spatiotemporal scales, and would seem to have very different causal effects on the distribution and form of cultural variants. Distinguishing, characterising, and organising various kinds of ecological factors of attraction seems a pressing issue for future CAT work.

I suggested above that transformative approaches implicate a background system, and that given the nature and state of such a background system, the same input may be transformed into different outputs. We can here apply this claim to the cultural realm and cash it out in more detail. Different factors of attraction determine the make-up of the background system against which transformations of ideational variants occur. I have here shown that there are at least three non-overlapping sets of factors of attraction that cultural epidemiologists appeal to when characterising the nature of this background system. I have also suggested that the theoretical role of ecological factors of attraction within this picture may require further scrutiny by cultural epidemiologists, insofar as the environment may play a variety of roles in causing change in the distribution and form of culture over time.

## Conclusion

CAT is a transformative approach to studying culture, one that should not be collapsed into preservative ones. CAT gains empirical and explanatory purchase on cultural phenomena by means of a number of idiosyncratic conceptual tools. I have here argued that these conceptual tools require further attention and clarification. In particular, I have shown that one can identify four distinct notions, or conceptualisations of the cultural attractor concept. These four notions—TRANSFORMATION PATTERN, CLUSTER POINT, EQUILIBRIUM POINT, and INCREASING FREQUENCY—pick out different kinds of populational tendencies. While in some cases these tendencies may lead to similar or identical variant distributions, this is likely only to occur in idealised scenarios. However, when de-idealised, for instance with the addition of sampling biases, these four notions may not coincide.

Along the same lines, I have shown that cultural epidemiologists appeal to a range of ‘factors of attraction’. Here I argued that these factors are not adequately captured by existing taxonomies, and pointed to areas where further conceptual work is required.

Indeed, there are a range of outstanding issues yet to be addressed in CAT. Should motivational attractors be identified with processes governing the selection of behaviours from a repertoire? How might ecological factors of attraction be taxonomised? Are there reasons to privilege some kinds of factors over others? I leave these questions for further analyses.

## Footnotes

1 While there seems to be a coalescing around the label of ‘cultural attractor theory’, or ‘cultural attraction theory’, to group together the work of individuals such a Dan Sperber, Nicolas Claidière, Olivier Morin, and others, researchers in the field still refer to their approach as ‘cultural epidemiology’, or the ‘epidemiological approach to culture’, and identify themselves as ‘cultural epidemiologists’ (Sperber 1985, 1996). While I prefer CAT as a label for picking out the theoretical commitments of this group of researchers, I will use ‘cultural epidemiologists’ to identify the researchers themselves.

2 Cf. Eriksson and Coultas (2009), Claidière and Whiten (2012), Lewens (2015), and Acerbi et al. (2016) for some recent criticisms and extensions of Henrich’s notion of conformist bias.

3 It is important to note that what may be best captured as a preservative process at one grain of analysis may not be so at a more fine-grained level (Acerbi and Mesoudi 2015). One of the more contentious issues between cultural epidemiologists and their interlocutors (e.g. Sperber 1996, 2000; Henrich and Boyd 2002; Henrich et al. 2008; Sperber and Claidière 2008) has been the extent to which learning is adequately characterised as preservative. The issue seems to hinge on an equivocation in the use of the term ‘learning’, with the cultural epidemiologists focusing on fine-grained details of single learning episodes, with researchers like Boyd, Henrich, and Richerson tending to abstract away from such detail and to operationalise learning as the acquisition of a model’s variant (Henrich et al. 2008; Henrich 2016).

4 Of course, this is perfectly compatible with it being the case that certain variants are acquired and manifested with a high degree of reliability from generation to generation. It is not necessary that transformative approaches deny the reliable recurrence of traits over time. It is just that transformative approaches would deny that reliable recurrence is best explained in terms of high-fidelity replication, or copying.

5 I have here distinguished between ‘approaches’ rather than ‘processes’ to suggest that whether or not something is seen as involving copying and error, rather than background systems and reconstructions, is sometimes a matter of the particular assumptions at work in specific cultural evolutionary models. But this is consistent with there being a determinate matter of fact as to whether the *processes* involved are best characterised as transformative or preservative.

6 It should be noted that Mesoudi et al.’s (2013) definition represents a shift from the initial articulation of guided variation as given by Boyd and Richerson (1985). For Boyd and Richerson, the subsequent modification of phenotypes in guided variation was taken to involve trial-and-error experimentation (p. 35. For an analogous articulation, cf. Henrich 2001, p. 996).

7 It should be noted that while some cultural attractors might change over ‘historical time’, it is not clear why Claidière and Sperber (2007) commit themselves to the claim that cultural attractors change at such a rate. This is an unusual claim, and one might think it is in conflict with the explanatory aims of CAT. If cultural attractors are supposed to play a part in providing distribution explanations for human culture, then the concept should be apt for explaining phenomena that are stable in the short term (fashions, fads, etc.). Since not all cultural epidemiologists seem to hold on to this claim about the long-lived nature of cultural attractors, Claidière and Sperber owe us an explanation for why cultural attractors, as they understand them, only change over ‘historical time’.

8 Following standard convention, I shall refer to distinct notions by using capital letters. Thus, ‘the notion of *xyz*’ is abbreviated to ‘*XYZ*’.

9 One may be tempted to argue that CLUSTER POINT and EQUILIBRIUM POINT are the *actual* cultural attractors, while TRANSFORMATION PATTERN and INCREASING FREQUENCY are mere tendencies *toward* such points (cf. Claidière et al. 2014, p. 4). Even if it makes sense in some cases to speak of patterned trajectories toward determine points, both CLUSTER POINT and EQUILIBRIUM POINT implicate distinct kinds of statistical tendencies – tendencies of populations to end up with certain kinds of frequency distributions. As I show below, because of this fact, it seems likely that these conceptualisations pick out very different kinds of tendencies from those involved in TRANSFORMATION PATTERN and INCREASING FREQUENCY. If this is true, then it is not clear what is gained by making the semantic gambit to distinguish ‘points’ and ‘patterns’, as one is still left with four distinct tendencies to account for.

10 Cultural epidemiologists also discuss (though not to as great an extent) the way in which the *production* of behaviour may also involve inferences (e.g. Sperber 1996; Morin 2015). I take it the idea is that one needs to know not only *how* to produce a behaviour but *when* it is appropriate to do so. Determining the *when* of behavioural expression might involve making inferences about the appropriateness of behaviour, and these inferences can be biased in various ways. In what follows, I use the label of reconstructive learning to capture these phenomena, together with the inferential biasing involved in reconstructive learning.
